# Trends in Light and Temperature Sensitivity Recommendations among Licensed Biotechnology Drug Products

**DOI:** 10.1007/s11095-023-03494-0

**Published:** 2023-04-06

**Authors:** Jennifer J. Kim, Jordan D. Pritts, Mai Ngo, Corey R. Estoll, V. Ashutosh Rao

**Affiliations:** 1 Office of Biotechnology Products, Center for Drug Evaluation and Research, Food and Drug Administration, Silver Spring, MD USA; 2 Laboratory of Applied Biochemistry, Division of Biotechnology Review and Research III, Office of Biotechnology Products, Center for Drug Evaluation and Research, Food and Drug Administration, 10903 New Hampshire Ave / Bldg. 52/72 Rm 2212, Silver Spring, MD 20993 USA; 3 Virginia Commonwealth University School of Pharmacy, Richmond, VA USA

**Keywords:** biotechnology, formulation, photodegradation, photostability, protein

## Abstract

**Purpose:**

Inherent structural and functional properties of biotechnology-derived therapeutic biologics make them susceptible to light- and temperature-induced degradation and consequently can influence their quality. Photosensitivity of therapeutic proteins continues to be examined, but the commonalities and trends of storage conditions and information about light and temperature sensitivity among currently licensed therapeutic proteins has not been previously surveyed.

**Methods:**

Using a comprehensive and relational database approach, we conducted a scientific survey of all licensed biotechnology-derived drug products with the goal of providing evidence-based information about recommended storage conditions of formulations sorted by light- and temperature-related attributes as described for each product at licensure.

**Results:**

We report the prevalence of indications for light and temperature sensitivity in formulations categorized by their presentation type, number of doses, container type, dosage form and active molecule type. We also report the storage temperature range across formulations and diluents for reconstitution and dilution. Formulations with excipients that potentially facilitate light-induced and thermal degradation were also noted.

**Conclusions:**

The result of our analysis indicates that light and temperature sensitivity are prevalent across therapeutic protein formulations. However, when a formulation is reconstituted or diluted, both light and temperature sensitivity are less clear. In addition, light and temperature sensitivity are more well defined in liquid formulations than lyophilized powder formulations, and more well defined in products manufactured in autoinjectors, prefilled-syringes, and pens than products in vials. Overall, our report provides a data-driven summary of storage conditions among therapeutic protein formulations to support the development of future biologic drug products.

## Introduction

Therapeutic protein drugs are an important class of medicine that are used for a broad range of indications as first line and combination therapies. The majority of therapeutic proteins are biotechnology-derived products that cannot be completely synthesized by chemical processes, and must be manufactured from biological sources including human, animal, plant, or microbial expression systems. Their large molecular size, post-translational modifications, and variety of biological materials involved in the manufacturing process all add to the complexity of these therapeutic proteins and their quality. For this reason, they are carefully characterized, monitored, controlled and stabilized to deliver the intended quality, safety, and efficacy profile [1].

Like all proteins, therapeutic protein drugs are susceptible to light-induced degradation, which can adversely impact their structure and function, and consequently the safety and quality of the drug products [2–4]. The light-induced degradation of therapeutic proteins has been reported for the exposure of formulations to UV and visible light, which can naturally happen during production, packing, storage, preparation, and administration [5]. The light-induced degradation can lead to not only changes in the appearance of a drug product, such as cloudiness, color change, or visible particle formation, but also changes in potency or adverse immunogenic responses [6]. Therefore, the temperature and storage conditions of therapeutic proteins must be appropriately monitored and controlled, posing a continuous challenge in manufacturing and packaging as well as transport and storage.

In the past few decades, there have been advancements in analytical sciences that have helped profile the photodegradation and thermal stress pathways of proteins. However, several therapeutic proteins have been marketed since the 1960s. Hence, there is a need to bridge the knowledge between the analytical characterization of the protein molecules at the time of approval, several decades earlier in some instances, to more recent risk assessments on light and temperature sensitivity under pharmaceutically relevant conditions. Additionally, labeling and package inserts that carry instructions for handling and use have also evolved in the past few decades. In alignment with the International Council for Harmonization (ICH) guideline for Stability Testing of new Drug Substances and Products, storage statements on labeling are based on the stability evaluation of the product. This includes photostability studies where samples are exposed to light and examined for any changes in physical properties and assayed for degradants by a method suitably validated for products likely to arise from photochemical degradation processes [3]. Subsequently, current research literature suggests a need to modernize the stability test conditions used by drug developers to mimic light and heat-mediated stress to more pharmaceutically and biologically appropriate conditions that are appropriate for protein macromolecules and consistent with our current understanding of relevant degradation pathways to maintain a drug supply that is safe and of high quality.

The impact of light exposure on a therapeutic protein with the role of excipients and package components is continued to be studied across the biotechnology industry [3]. Existing resources and literature, however, rarely extend to how product recommendations are made for storage temperature or light exposure in the context of different formulation attributes or diluents used, conditions of use across different climate zones, and whether they are product-specific or dosage form-specific. There is still a need for additional clarity on scientifically appropriate and pharmaceutically-relevant studies to characterize the degradation profile of biologic based drugs regarding their photostability and thermal stability.

In this report, we present a comprehensive survey of formulations for all currently approved or licensed biotechnology-derived therapeutic protein drugs to analyze trends in storage conditions and indications for temperature and light sensitivity on product labeling in relation to different formulation attributes. The terms “indication” and “no indication” in this paper refer to the presence or absence of storage instructions to protect the formulation from light and not to freeze on the product labeling. We aim to identify any commonalities and extrapolate information on various pharmaceutically relevant aspects, including multiple formulation attributes, active pharmaceutical ingredient (API) types, dosage form, intended number of doses (single-dose or multiple-dose), container type, diluents, storage temperature range, and excipients. Our goal is to provide insight on formulations used in marketed therapeutic proteins in the context of temperature and light sensitivity and improve evidence-based risk assessment for the development of future therapeutic protein drugs and the instructions for use they carry upon approval. Additionally, we anticipate that such data will aid the development of analytical test methods, relevant test conditions and candidate protein therapeutics with greater photo and thermal stability under a wide range of storage and in-use conditions.

## Materials and Methods

### Data Sources

To begin curating a database of therapeutic protein drug formulations, a list of drug products currently approved or licensed by the Office of Biotechnology Products in the Office of Pharmaceutical Quality, Center for Drug Evaluation and Research (CDER) was generated. CDER’s list of approved or licensed biological products was matched with the Purple Book or the List of Approved New Drug Applications (NDAs) for biological products to confirm inclusion of transition and biosimilar products in the database [7, 8]. The current presentation, instructions for use, and composition for each drug product was obtained from current prescribing information, publicly available on the Drugs@FDA website [9]. If prescribing information (PI) was unavailable from the Drugs@FDA website, prescribing information provided by the drug manufacturer’s website was utilized as a secondary resource. The drug products, dosage forms, and instructions for light and temperature recommendations were analyzed using a relational database program, Microsoft Access (Microsoft Corporation, Office 365 ProPlus, Access version 1902), which allowed analysis of large data through a platform and a user interface for performing targeted queries [1].

### Data Curation and Survey Method

The data were organized by using the assigned National Drug Code (NDC) to identify unique formulations that differ in active ingredients, dosage form, number of doses and container type. Formulations that had the same formulation attributes but differ in package size were counted as a single entry for the analysis to minimize duplication of data. For each unique formulation that was identified, excipients and storage requirements including the temperature range, storage duration, indication to protect from light, and not to freeze was recorded. Products supplied as lyophilized powder require a reconstitution step by adding a solvent to dissolve the powder and form a solution for parenteral administration. Products supplied as solution may undergo an additional dilution process by adding a diluent to reduce the concentration before administration. For formulations that were further reconstituted or diluted before administration, the specific storage requirements were recorded with the type of solution used.

Solutions used for reconstitution or dilution were categorized into five broad categories including sterile water, sodium chloride, dextrose, Ringer’s injection and formulation-specific buffer. Sterile water includes Sterile Water for Injection, USP with different types of additives including benzyl alcohol, glycerin, mannitol, metacresol, poloxamer 188, or a combination. Sodium chloride includes concentrations of 0.54% w/v, 0.6% w/v and 0.9% w/v Sodium Chloride Injection, USP with additives including benzyl alcohol, calcium chloride dihydrate, or albumin. Dextrose includes concentration of 5% w/v and 10% w/v, and Ringer’s injection includes Ringer’s lactate solution as well. Formulation specific buffer was specific to each product and the composition was described in the product labeling, which included a combination of buffers. For the purposes of the analysis, APIs were categorized into eight different types including monoclonal antibodies, antibody conjugates, enzymes, cytokines and growth factors, polypeptide hormones, pulmonary surfactants (phospholipoproteins that reduce alveolar surface tension), recombinant fusion proteins, and toxins. Antibody conjugates molecule types included antibody drug conjugates, antibody linker-chelator conjugates and antibody-toxin conjugates.

### Statistical Analysis

The number of formulations with indications to protect from light and not to freeze was counted to calculate the prevalence of these indications. For each formulation attribute surveyed, the number of formulations in each category was counted and prevalence of light and temperature sensitivity indications were calculated within each category for comparison among the different categories. The same analysis was repeated for reconstituted and diluted formulations separately. Analyses for dosage form and excipients were not repeated for reconstituted or diluted formulations, because liquid is the only dosage form for these formulations and excipients do not change with the change in presentation. Formulations with the same recommended storage temperature range were counted and prevalence was counted using the total number of formulations in each presentation status category. Graphs were generated using Prism 6 for Windows v6.05 (GraphPad Software, LaJolla, CA).

## Results

### Comprehensive survey of formulations with light and temperature sensitivity

**Fig. 1:**
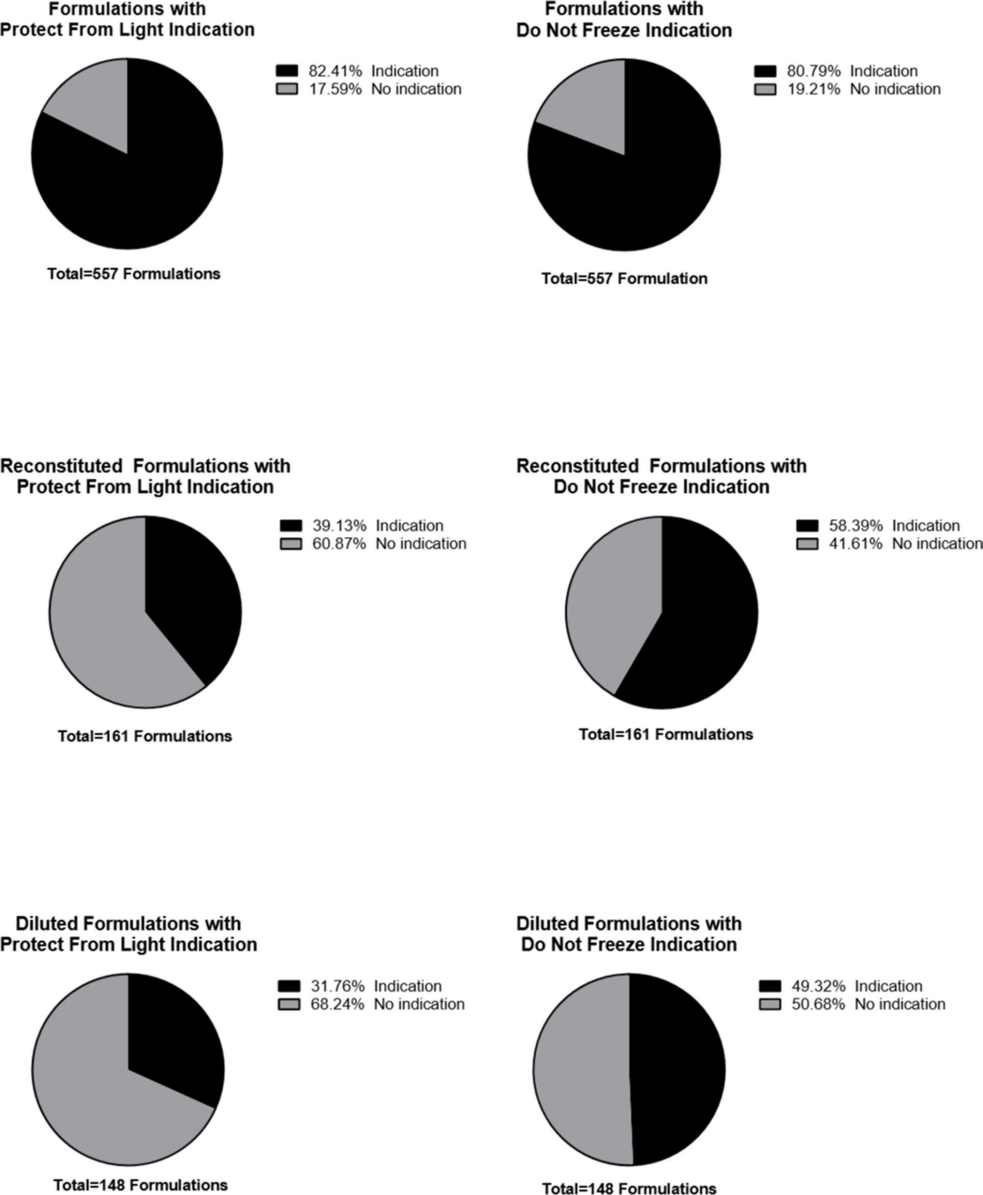
Prevalence of Formulations with Light and Temperature Sensitivity Indication.

As of August 2022, there are 226 biotechnology-derived APIs currently approved by CDER and marketed in the U.S. in 767 different formulations. Of the 767 original formulations, 557 were considered unique based on having a unique combination of API, number of doses, container type, or dosage form. Out of 557 unique formulations, 459 formulations (82.41%) had an indication to protect from light and 450 formulations (80.79%) had an indication not to freeze (Fig. 1). 407 formulations (73.07%) had both indications while 52 formulations (9.34%) had an indication to protect from light without an indication not to freeze, and 43 formulations (7.72%) had an indication not to freeze without an indication to protect from light (Table I). When formulations are further reconstituted or diluted for the indicated route of administration, there were fewer indications to protect the reconstituted or diluted solution from light compared to the as-supplied presentation. Only 63 formulations (39.13%) out of 161 reconstituted formulations and 47 formulations (31.76%) out of 148 diluted formulations had an indication to protect from light. A slightly higher proportion of reconstituted formulations had an indication not to freeze with 94 formulations (58.39%) and 73 diluted formulations (49.32%) had an indication not to freeze (Tables II and III).

**Table I:** Number of Formulations with Light and Temperature Sensitivity Indication

	Protect from Light	Do Not Freeze		
Indication	459 (82.41%)	450 (80.79%)		
No Indication	98 (17.59%)	107 (19.21%)		
Total Formulations	557	557		
		Protect from Light		Total Formulations
		Indication	No Indication	
Do Not Freeze	Indication	407 (73.07%)	43 (7.72%)	450
	No Indication	52 (9.34%)	55 (9.87%)	107
Total Formulations		459	98	557

**Table II:** Number of Reconstituted Formulations with Light and Temperature Sensitivity Indication

Reconstituted Formulations	Protect from Light	Do Not Freeze
Indication	63 (39.13%)	94 (58.39%)
No Indication	98 (60.87%)	67 (41.61%)
Total Formulations	161	161

**Table III:** Number of Diluted Formulations with Light and Temperature Sensitivity Indication

Diluted Formulations	Protect from Light	Do Not Freeze
Indication	47 (31.76%)	73 (49.32%)
No Indication	101 (68.24%)	75 (50.68%)
Total Formulations	148	148

**Fig. 2:**
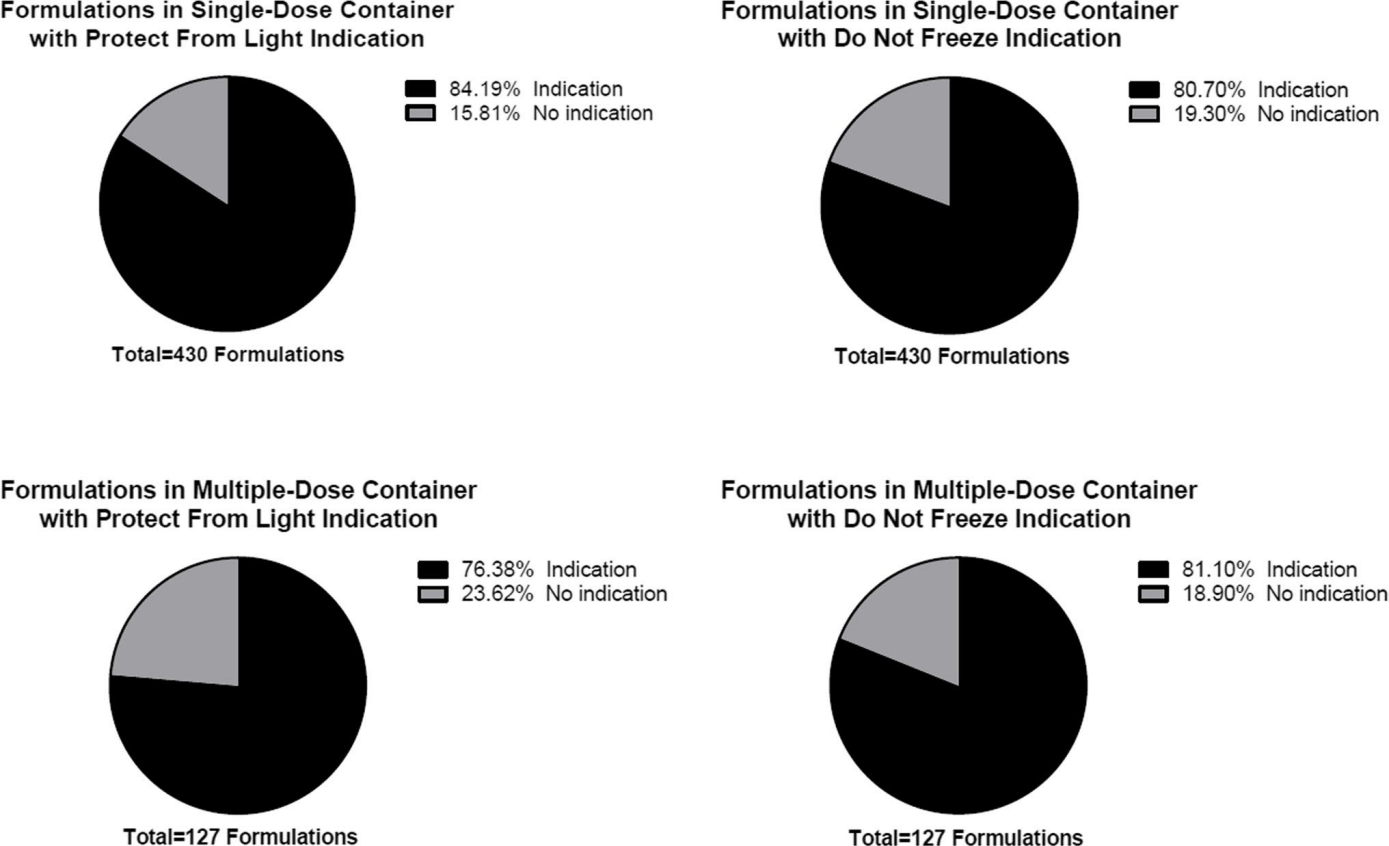
Prevalence of Formulations with Light and Temperature Sensitivity Indication by Number of Doses.

**Fig. 3:**
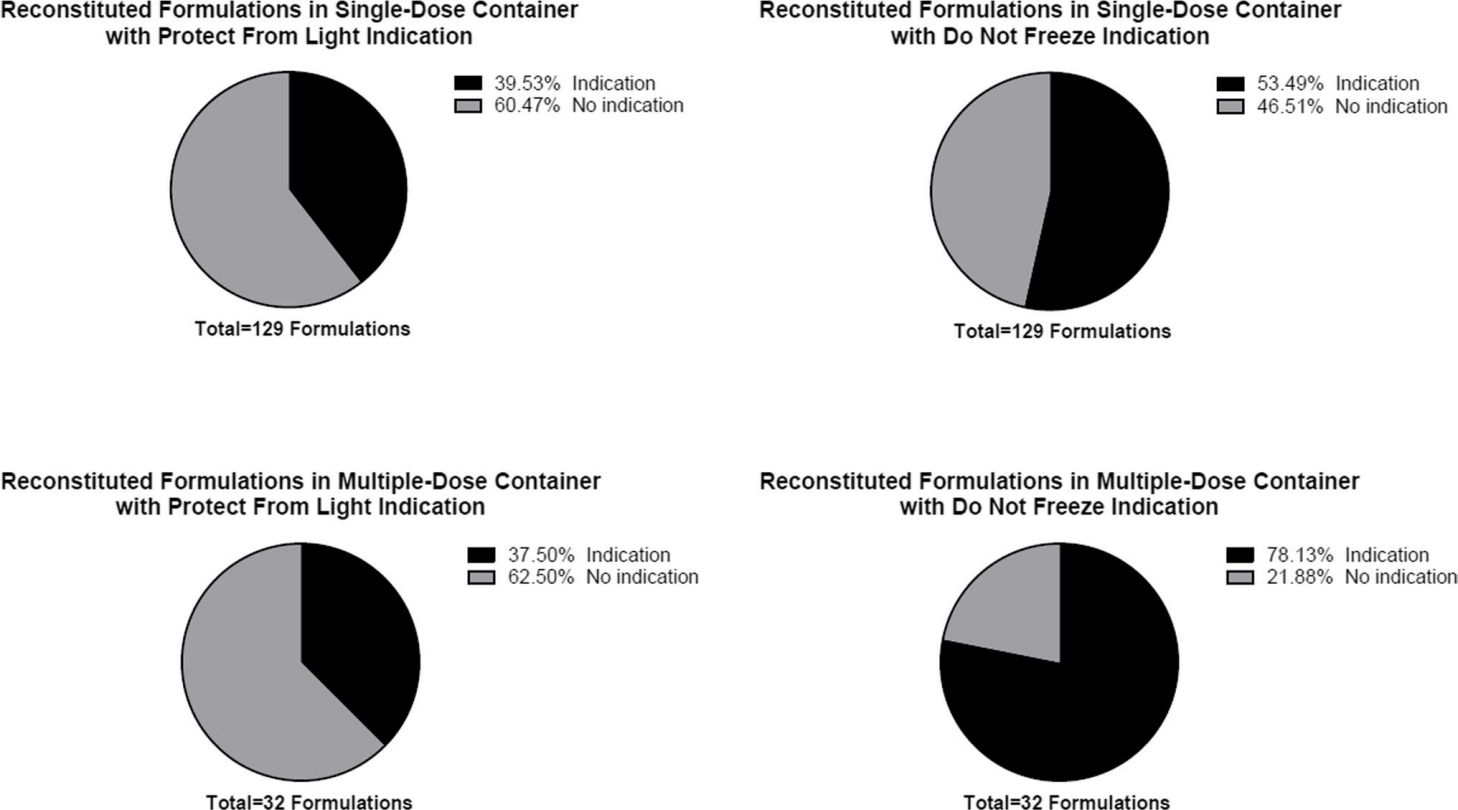
Prevalence of Reconstituted Formulations with Light and Temperature Sensitivity Indication by Number of Doses.

**Fig. 4:**
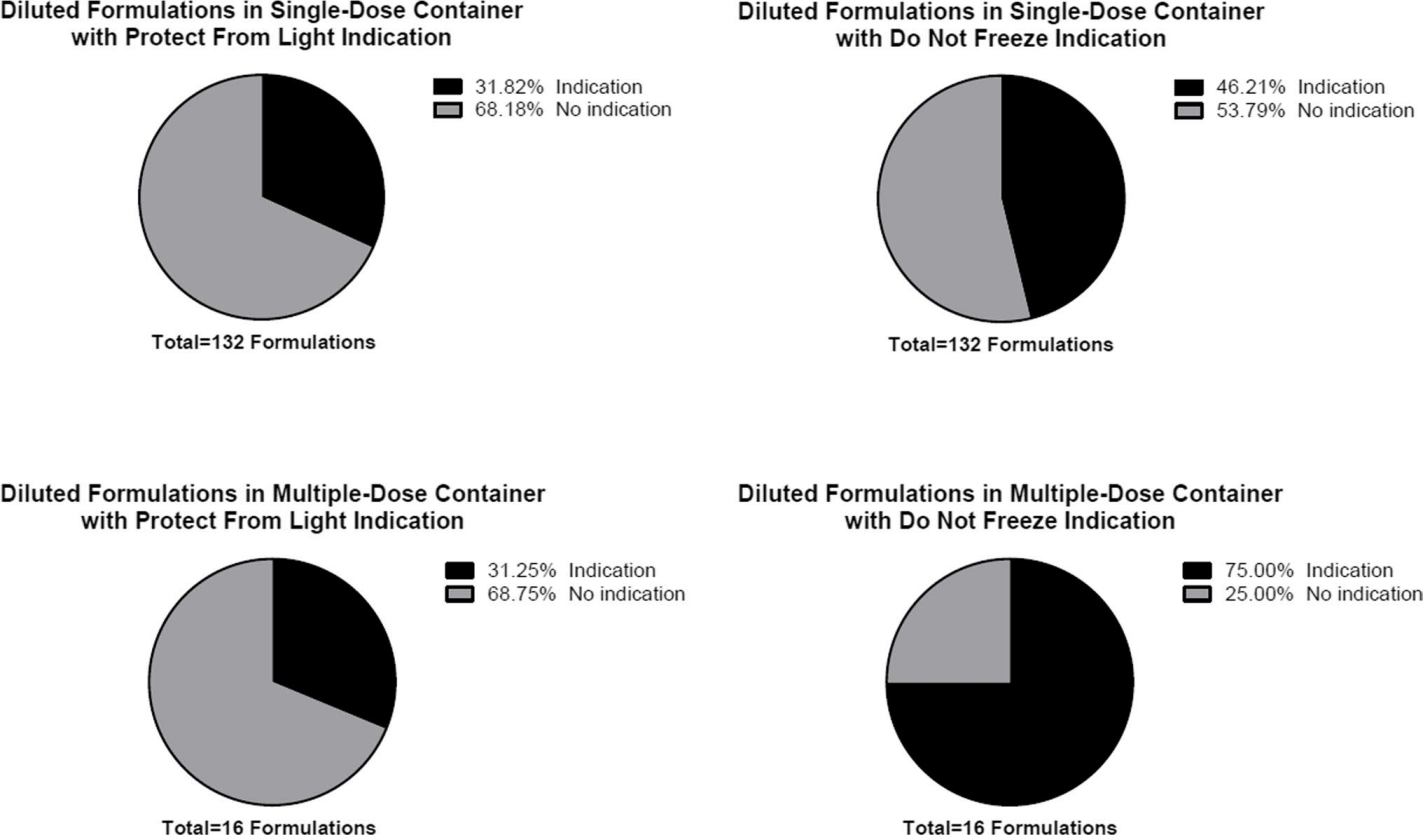
Prevalence of Diluted Formulations with Light and Temperature Sensitivity Indication by Number of Doses.

Among 557 formulations, 430 formulations (77.20%) were manufactured to deliver a single dose, while 127 formulations (22.80%) were manufactured to deliver multiple doses. Of 430 single-dose formulations, 362 formulations (84.19%) had indications to protect from light and 347 formulations (80.70%) had indications not to freeze. Similarly for multiple-dose formulations, 97 formulations (76.38%) had indications to protect from light and 103 formulations (81.10%) had indications not to freeze (Table IV and Fig. 2). When a formulation required reconstitution, 51 out of 129 single-dose formulations (39.53%) had an indication to protect from light and 69 formulations (53.49%) had an indication not to freeze. For multiple-dose formulations, 12 out of 32 formulations (37.50%) had a light sensitivity indication while a higher number of formulations had a temperature sensitivity indication at 25 out of 32 (78.13%) (Table V and Fig. 3). A similar prevalence was observed for diluted formulations with 31.82% single-dose formulations and 31.25% multiple-dose formulations having an indication to protect from light and 46.21% single-dose formulations and 75.00% multiple-dose formulations having an indication not to freeze (Table VI and Fig. 4).

**Table IV:** Number of Formulations with Light and Temperature Sensitivity Indication by Number of Doses

Number of Doses	Total Formulations (n = 557)	Protect from Light		Do Not Freeze	
		Indication	No Indication	Indication	No Indication
Single-dose	430 (77.20%)	362 (84.19%)	68 (15.81%)	347 (80.70%)	83 (19.30)
Multiple-dose	127 (22.80%)	97 (76.38%)	30 (23.62%)	103 (81.10%)	24 (18.90%)

**Table V:** Number of Reconstituted Formulations with Light and Temperature Sensitivity Indication by Number of Doses

Number of Doses	Total Formulations (n = 161)	Protect from Light		Do Not Freeze	
		Indication	No Indication	Indication	No Indication
Single-dose	129 (80.12%)	51 (39.53%)	78 (60.47%)	69 (53.49%)	60 (46.51%)
Multiple-dose	32 (19.88%)	12 (37.50%)	20 (62.50%)	25 (78.13%)	7 (21.88%)

**Table VI:** Number of Diluted Formulations with Light and Temperature Sensitivity Indication by Number of Doses

Number of Doses	Total Formulations (n = 148)	Protect from Light		Do Not Freeze	
		Indication	No Indication	Indication	No Indication
Single-dose	132 (89.19%)	42 (31.82%)	90 (68.18%)	61 (46.21%)	71 (53.79%)
Multi-dose	16 (10.81%)	5 (31.25%)	11 (68.75%)	12 (75.00%)	4 (25.00%)

**Fig. 5:**
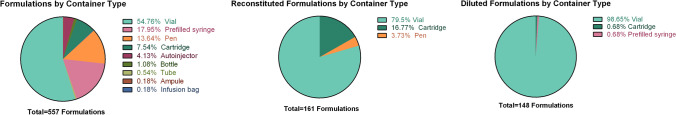
Prevalence of Formulations by Container Type.

In addition to the number of intended doses, we surveyed types of containers used for the manufacture of therapeutic protein drugs. There were nine different container types, including ampule, autoinjector, bottle, cartridge, infusion bag, pen, prefilled syringe, tube, and vial. Glass vial was the most prevalent container type used in 305 formulations (54.76%), followed by 100 formulations in prefilled syringe (17.95%) and 76 formulations in pen (13.64%) (Fig. 5). Out of 305 formulations available in vials, 228 formulations (74.75%) had an indication to protect from light and 221 formulations (72.46%) had an indication not to freeze (Table VII). For most of the container types, there were more formulations that had light and temperature sensitivity indication, except for the ones manufactured in bottles or tubes. For formulations manufactured in autoinjector and prefilled syringe, all 23 autoinjector and 100 prefilled syringe formulations had an indication to protect from light, and all autoinjector formulations also had an indication not to freeze (Table VII). Formulations requiring reconstitution and dilution were mostly manufactured in vials, while a smaller proportion was formulated in pens and cartridges. Fewer formulations had an indication to protect from light and not to freeze when reconstitution and dilution were required (Tables VIII and IX).

**Table VII:** Number of Formulations with Light and Temperature Sensitivity Indication by Container Type

Container Closure	Total Formulations (n = 557)	Protect from Light		Do Not Freeze	
		Indication	No Indication	Indication	No Indication
Ampule	1 (0.18%)	1 (100.0%)	0 (0.00%)	0 (0.00%)	1 (100.0%)
Autoinjector	23 (4.13%)	23 (100.0%)	0 (0.00%)	23 (100.0%)	0 (0.00%)
Bottle	6 (1.08%)	1 (16.67%)	5 (83.33%)	0 (0.00%)	6 (100.0%)
Cartridge	42 (7.54%)	35 (83.33%)	7 (16.67%)	36 (85.71%)	6 (14.29%)
Infusion bag	1 (0.18%)	1 (100.0%)	0 (0.00%)	1 (100.0%)	0 (0.00%)
Pen	76 (13.64%)	70 (92.11%)	6 (7.89%)	76 (100.0%)	0 (0.00%)
Prefilled syringe	100 (17.95%)	100 (100.0%)	0 (0.00%)	92 (92.00%)	8 (8.00%)
Tube	3 (0.54%)	0 (0.00%)	3 (100.0%)	1 (33.33%)	2 (66.67%)
Vial	305 (54.76%)	228 (74.75%)	77 (25.25%)	221 (72.46%)	84 (27.54%)

**Table VIII:** Number of Reconstituted Formulations with Light and Temperature Sensitivity Indication by Container Type

Container Closure	Total Formulations (n = 161)	Protect from Light		Do Not Freeze	
		Indication	No Indication	Indication	No Indication
Cartridge	27 (16.77%)	17 (62.96%)	10 (37.04%)	18 (66.67%)	9 (33.33%)
Pen	6 (3.73%)	0 (0.00%)	6 (100.0%)	6 (100.0%)	0 (0.00%)
Vial	128 (79.50%)	46 (35.94%)	82 (64.06%)	70 (54.69%)	58 (45.31%)

**Table IX:** Number of Diluted Formulations with Light and Temperature Sensitivity Indication by Container Type

Container Closure	Total Formulations (n = 148)	Protect from Light		Do Not Freeze	
		Indication	No Indication	Indication	No Indication
Cartridge	1 (0.69%)	1 (100.0%)	0 (0.00%)	1 (100.0%)	0 (0.00%)
Prefilled syringe	1 (0.69%)	0 (0.00%)	1 (100.0%)	0 (0.00%)	1 (100.0%)
Vial	146 (98.65%)	46 (31.51%)	100 (68.49%)	72 (49.32%)	74 (50.68%)

**Fig. 6:**
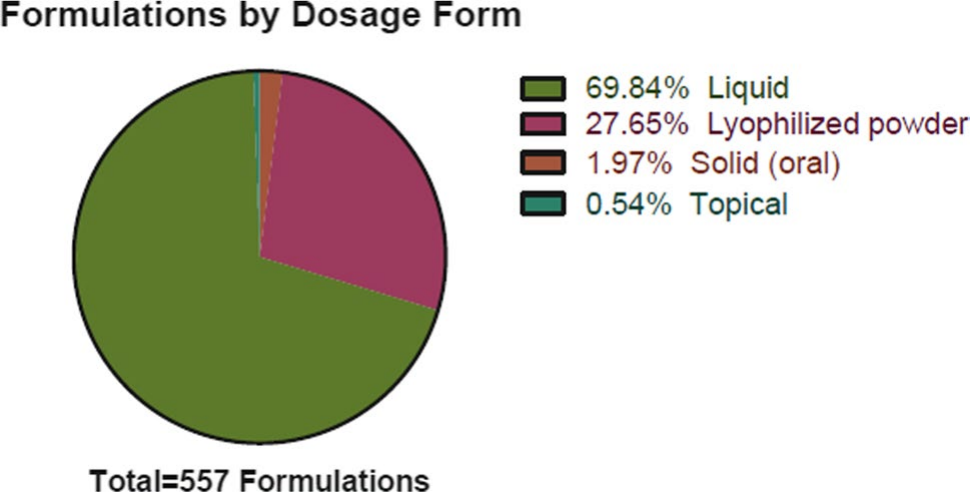
Prevalence of Formulations by Dosage Form.

A similar trend was observed when formulations were surveyed by dosage form. The majority of formulations are manufactured in a liquid dosage form (69.84%), and over 90% of liquid formulations had an indication to protect from light (95.37%) and not to freeze (93.32%) (Table X and Fig. 6). For lyophilized powder formulations that constituted 27.65% of all formulations, about half of the products had an indication to protect from light (57.14%) and not to freeze (55.84%) (Table X). Most solid and topical dosage forms did not have any light or temperature sensitivity recommendation.

**Table X:** Number of Formulations with Light and Temperature Sensitivity Indication by Dosage Form

Dosage From	Total Formulations (n = 557)	Protect from Light		Do Not Freeze	
		Indication	No Indication	Indication	No Indication
Solid (oral)	11 (1.97%)	0 (0.00%)	11 (100.0%)	0 (0.00%)	11 (100.0%)
Lyophilized powder	154 (27.65%)	88 (57.14%)	66 (42.86%)	86 (55.84%)	68 (44.16%)
Liquid	389 (69.84%)	371 (95.37%)	18 (4.63%)	363 (93.32%)	26 (6.68%)
Topical	3 (0.54%)	0 (0.00%)	3 (100.0%)	1 (33.33%)	2 (66.67%)

**Fig. 7:**
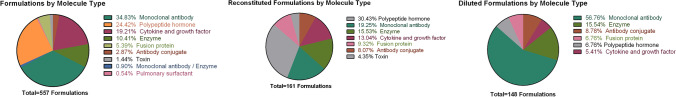
Prevalence of Formulations by Molecule Type.

Lastly, formulations were surveyed based on molecule type of the APIs, categorized into eight different types including antibody conjugate, cytokine and growth factor, enzyme, monoclonal antibody, polypeptide hormone, pulmonary surfactant, fusion protein, and toxin. One additional category was included in the survey for monoclonal antibody and enzyme combination products which consist of 5 formulations. Among the different molecule type categories, monoclonal antibody was the most prevalent with 194 formulations (34.83%), followed by 136 formulations (24.42%) of polypeptide hormone, 107 formulations (19.21%) of cytokine and growth factor, and 58 formulations (10.41%) of enzyme (Fig. 7). For most molecule types, there were more formulations that had light and temperature sensitivity indication, except for enzymes and toxins where only 27 out of 58 enzyme formulations (46.55%) and 1 out of 8 formulations (12.50%) had indication not to freeze (Table XI). For formulations that require reconstitution, the most prominent molecule type was polypeptide with 49 formulations (30.43%) (Table XII). When comparing light and temperature sensitivity, indication to not freeze was more commonly found than indication to protect from light for both reconstituted and diluted formulations. (Tables XII and XIII).

**Table XI:** Number of Formulations with Light and Temperature Sensitivity Indication by Molecule Type

Molecule Type	Total Formulations (n = 557)	Protect from Light		Do Not Freeze	
		Indication	No Indication	Indication	No Indication
Antibody conjugate	16 (2.87%)	11 (68.75%)	5 (31.25%)	14 (87.50%)	2 (12.50%)
Cytokine and growth factor	107 (19.21%)	83 (77.57%)	24 (22.43%)	89 (83.18%)	18 (16.82%)
Enzyme	58 (10.41%)	30 (51.72%)	28 (48.28%)	27 (46.55%)	31 (53.45%)
Monoclonal antibody	194 (34.83%)	179 (92.27%)	15 (7.73%)	174 (89.69%)	20 (10.31%)
Monoclonal antibody/Enzyme	5 (0.90%)	5 (100.0%)	0 (0.00%)	5 (100.0%)	0 (0.00%)
Polypeptide hormone	136 (24.42%)	115 (84.56%)	21 (15.44%)	115 (84.56%)	21 (15.44%)
Pulmonary surfactant	3 (0.54%)	3 (100.0%)	0 (0.00%)	0 (0.00%)	3 (100.0%)
Fusion protein	30 (5.39%)	29 (96.67%)	1 (3.33%)	25 (83.33%)	5 (16.67%)
Toxin	8 (1.44%)	4 (50.00%)	4 (50.00%)	1 (12.50%)	7 (87.50%)

**Table XII:** Number of Reconstituted Formulations with Light and Temperature Sensitivity Indication by Molecule Type

Molecule Type	Total Formulations (n = 161)	Protect from Light		Do Not Freeze	
		Indication	No Indication	Indication	No Indication
Antibody conjugate	13 (8.07%)	9 (69.23%)	4 (30.77%)	13 (100.0%)	0 (0.00%)
Cytokine and growth factor	21 (13.04%)	2 (9.52%)	19 (90.48%)	18 (85.71%)	3 (14.29%)
Enzyme	25 (15.53%)	8 (32.00%)	17 (68.00%)	8 (32.00%)	17 (68.00%)
Monoclonal antibody	31 (19.25%)	8 (25.81%)	23 (74.19%)	19 (61.29%)	12 (38.71%)
Polypeptide hormone	49 (30.43%)	23 (46.94%)	26 (53.06%)	31 (63.27%)	18 (36.73%)
Fusion protein	15 (9.32%)	10 (66.67%)	5 (33.33%)	2 (13.33%)	13 (86.67%)
Toxin	7 (4.35%)	3 (42.86%)	4 (57.14%)	3 (42.86%)	4 (57.14%)

**Table XIII:** Number of Diluted Formulations Type with Light and Temperature Sensitivity Indication by Molecule Type

Molecule Type	Total Formulations (n = 148)	Protect from Light		Do Not Freeze	
		Indication	No Indication	Indication	No Indication
Antibody conjugate	13 (8.78%)	7 (53.85%)	6 (46.15%)	12 (92.31%)	1 (7.69%)
Cytokine and growth factor	8 (5.41%)	0 (0.00%)	8 (100.0%)	3 (37.50%)	5 (62.50%)
Enzyme	23 (15.54%)	10 (43.48%)	13 (56.52%)	12 (52.17%)	11 (47.83%)
Monoclonal antibody	84 (56.76%)	21 (25.00%)	63 (75.00%)	39 (46.43%)	45 (53.57%)
Polypeptide hormone	10 (6.76%)	5 (50.00%)	5 (50.00%)	6 (60.00%)	4 (40.00%)
Fusion protein	10 (6.76%)	4 (40.00%)	6 (60.00%)	1 (10.00%)	9 (90.00%)

Most therapeutic proteins are produced using recombinant DNA technology, which involves combination of DNA molecules from two different species to produce a wide range of peptides, proteins, and biochemicals [10]. Out of 226 APIs, 202 APIs are produced recombinantly while 24 APIs are considered naturally derived, consisting of 37 unique formulations. Of these 37 formulations with naturally derived APIs, 18 formulations (48.65%) have an indication to protect form light and 8 formulations (21.62%) have an indication not to freeze. There were 441 recombinant product formulations (84.81%) that had an indication to protect from light and 442 formulations (85.00%) had an indication not to freeze.

### Range of storage temperatures across products

Recommended storage temperature range was also surveyed in all presentation status of formulations. Most formulations were recommended to be stored refrigerated at 2°C to 8°C with 512 formulations (91.92%). 6 formulations (1.08%) were stored frozen, 24 formulations (4.31%) were stored at room temperature ranging from 9°C to 40°C, and 15 formulations (2.69%) were stored at either refrigerator or at room temperature ranging from 2°C to 30°C (Table XIV). For reconstituted and diluted solution, immediate use was often advised, and, if immediate use was not possible, a recommended storage temperature was provided with specific duration of time ranging from 1 h to 60 days. 112 formulations out of 161 reconstituted formulations (69.57%) and 139 formulations out of 273 diluted formulations (50.92%) were advised to be stored under refrigeration. Compared to formulations that are used as-supplied, a higher proportion of reconstituted and diluted formulations had recommendations to be stored at room temperature with 27 reconstituted formulations (16.77%) and 106 diluted formulations (38.83%) at a temperature range of 9°C to 27°C and duration of 3 h to 28 days (Table XIV).

**Table XIV:** Number of Formulations by Recommended Storage Temperature Range

Temperature Range	Formulation
Below 0°C (frozen)	6 (1.08%)
2°C—8°C	512 (91.92%)
2°C—25°C	9 (1.62%)
2°C—30°C	3 (0.54%)
3°C—25°C	3 (0.54%)
9°C—25°C	2 (0.36%)
20°C—25°C	10 (1.80%)
15°C—30°C	10 (1.80%)
15°C—40°C	2 (0.36%)
Total Formulations	557
Temperature Range	Reconstituted Formulations
2°C—8°C	112 (69.57%)
2°C—25°C	7 (4.35%)
2°C—27°C	1 (0.62%)
2°C—30°C	14 (8.70%)
9°C—25°C	8 (4.97%)
15°C—30°C	1 (0.62%)
20°C—25°C	17 (10.56%)
23°C—27°C	1 (0.62%)
Total Formulations	161
Temperature Range	Diluted Formulations
2°C—8°C	139 (50.92%)
2°C—20°C	1 (0.37%)
2°C—25°C	19 (6.96%)
2°C—27°C	2 (0.73%)
2°C—30°C	6 (2.20%)
9°C—25°C	31 (11.36%)
9°C—30°C	3 (1.10%)
15°C—25°C	9 (3.30%)
20°C—25°C	60 (21.98%)
23°C—27°C	3 (1.10%)
Total Formulations	273*

^*^The total number of formulations is larger than number of unique diluted formulations (148), because a formulation can be diluted with more than one diluent and can have multiple storage conditions

### Commonly used diluents for reconstitution and dilution with light and sensitivity indications

Sterile water for injection was the most prevalent diluent for reconstitution with 134 formulations (82.23%) out of 161 formulations, and 23 formulations (14.29%) were reconstituted with sodium chloride containing solution. Only 57 formulations (42.54%) reconstituted with sterile water for injection and 5 formulations (21.74%) reconstituted with sodium chloride had an indication to protect from light. 7 formulations (30.43%) reconstituted with sodium chloride had an indication not to freeze, but the proportion was much higher for formulations reconstituted with sterile water with 87 formulations (64.93%) (Table XV). Dilution process typically involved a larger volume of diluent than reconstitution, and most formulations used sodium chloride, dextrose and Ringer’s injection as diluents. 195 formulations (71.43%) used sodium chloride containing solution, 67 formulations (24.54.%) with dextrose, 10 formulations (3.66%) with Ringer’s injection and 1 formulation (0.37%) with formulation-specific buffer provided with the product. For formulations using sodium chloride as a diluent, 68 formulations (34.87%) had an indication to protect from light and 100 formulations (51.28%) had an indication not to freeze. Similar proportion was observed in other formulations using dextrose and Ringer’s injection as diluents (Table XVI).

**Table XV:** Number of Reconstituted Formulations by Diluent with Light and Temperature Sensitivity Indication

Diluent	Total Formulations (n = 161)	Protect from Light		Do Not Freeze	
		Indication	No Indication	Indication	No Indication
Sterile Water	134 (83.23%)	57 (42.54%)	77 (57.46%)	87 (64.93%)	47 (35.07%)
Sodium Chloride	23 (14.29%)	5 (21.74%)	18 (78.26%)	7 (30.43%)	16 (69.57%)
Dextrose	2 (1.24%)	1 (50.00%)	1 (50.00%)	0 (0.00%)	2 (100.0%)
Formulation-Specific Buffer	2 (1.24%)	0 (0.00%)	2 (100.0%)	0 (0.00%)	2 (100.0%)

**Table XVI:** Number of Diluted Formulations by Diluent with Light and Temperature Sensitivity Indication

Diluent	Total Formulations (n = 273)	Protect from Light		Do Not Freeze	
		Indication	No Indication	Indication	No Indication
Sodium Chloride	195 (71.43%)	68 (34.87%)	127 (65.13%)	100 (51.28%)	95 (48.72%)
Dextrose	67 (24.54%)	16 (23.88%)	51 (76.12%)	30 (44.78%)	37 (55.22%)
Ringer’s Injection	10 (3.66%)	3 (30.00%)	7 (70.00%)	6 (60.00%)	4 (40.00%)
Formulation-Specific Buffer	1 (0.37%)	0 (0.00%)	1 (100.0%)	1 (100.0%)	0 (0.00%)

### Comparison of storage requirements among biosimilar formulations

Comparisons of storage information among biosimilar formulations were conducted to gain insight on the complexity of manufacturing processes leading to differences in highly similar formulations. By definition, a biosimilar is a biological product that is highly similar to an existing approved or licensed product by the structure and function, and has no clinically meaningful difference in terms of safety, purity, and potency [11]. A biosimilar product can include minor differences in clinically inactive components, and this can lead to differences in the storage requirements. There were 58 formulations that were biosimilar, and 52 biosimilar formulations (89.66%) had the same storage requirements as the reference product including the recommended temperature range, indication to protect from light and not to freeze. Out of the 6 biosimilar formulations that had different storage instructions when compared to the innovator product, 4 formulations had addition of light sensitivity indication and 2 formulations had temperature sensitivity indication.

### Possible enhancement of light-induced degradation with interactions among excipients and pH

Excipients are a critical component of a drug product which enhances the manufacturability, stability, and delivery of the drug product [1]. Hence the presence of specific excipients is a key risk assessment criterion for light and temperature sensitivity of protein products. There are studies that have evaluated the effect of excipients on light-induced degradation and interactions among the excipients that can lead to unforeseen outcomes [12, 13]. Excipients in therapeutic protein formulations can both promote or inhibit light-induced degradation pathways in pharmaceutical buffers. For instance, citrate buffer under the right circumstances can promote oxidative degradation of these proteins under exposure to UV and visible light [12]. In this study, addition of selected amino acids or carbohydrates in citrate buffer enhanced the oxidation yields, specifically methionine sulfoxide. EDTA could also enhance oxidation of formulation constituents under light exposure. Surveying the formulations with their excipients, 59 formulations were identified to have citrate buffer in their formulation. Of these 59 formulations with citrate buffer, 44 formulations also contained either arginine, lysine, histidine, mannitol, sucrose, trehalose or EDTA, which were studied and found to elevate the risk of higher oxidation yields in citrate buffer. In regard to light sensitivity indication, 51 formulations (86.44%) out of 59 formulations had an indication to protect from light. Out of 8 formulations (13.56%) without an indication to protect from light, 1 formulation contained mannitol, 1 formulation contained trehalose and 1 formulation contained EDTA.

To identify other excipients that may correlate with light or temperature sensitivity, a correlative database search was conducted. The proportion of formulations containing each excipient with light and temperature sensitivity indications were tabulated. Excipients that were found in five or more formulations were examined and a heat map was generated to identify excipients that correlate with light and temperature sensitivity indications (Table XVII). The midpoint average for the heat map was set to the overall prevalence for all formulations at 82.41% for light sensitivity and 80.79% for temperature sensitivity. Excipients that resulted in higher light or temperature sensitivity indications than the overall average were colored red while excipients that resulted in lower-than-average indications were colored blue. Percentages that deviated from the average with a p value lower than 0.05 via a binomial test are bolded and underlined for reference. Formulations containing sodium chloride, sodium citrate, metacresol, glycerin, methionine, and zinc oxide had increased instances of both light and temperature sensitivity indications than the overall average. Formulations that contained phosphoric acid had a decreased occurrence of both light and temperature sensitivity indications. Histidine, citric acid, sorbitol, arginine, poloxamer 188, and acetate containing formulations had higher rates of indications to protect from light without significant differences in rates of indications not to freeze when compared to the overall average. Sodium phosphate monobasic, human albumin, sodium phosphate tribasic, and sodium succinate had lower occurrence of light sensitivity than average with no significant difference in occurrence of temperature sensitivity. Polysorbate 80 and hydrochloric acid containing formulations had increased temperature sensitivity while succinic acid and sodium containing formulations showed decreased temperature sensitivity without a significant difference in light sensitivity. In Table XVIII, the effects of formulation pH on light and temperature sensitivity were investigated. A total of 352 formulations expressed pH information on their product labels. 325 (92.33%) formulations had a protect from light indication while 319 (90.63%) had a do not freeze indication. Based on the data analyzed, the pH of a formulation did not have any statistically significant correlation with a do not freeze indication. Acidic formulations (between pH 1–5.99) had a higher-than-average occurrence of protect from light indications (97.01%) when compared to formulations at more physiological conditions from pH 6–8 (89.40%). Only 1 formulation had a pH between 8.01–14 and had both protect from light and do not freeze indications.

**Table XVII:**
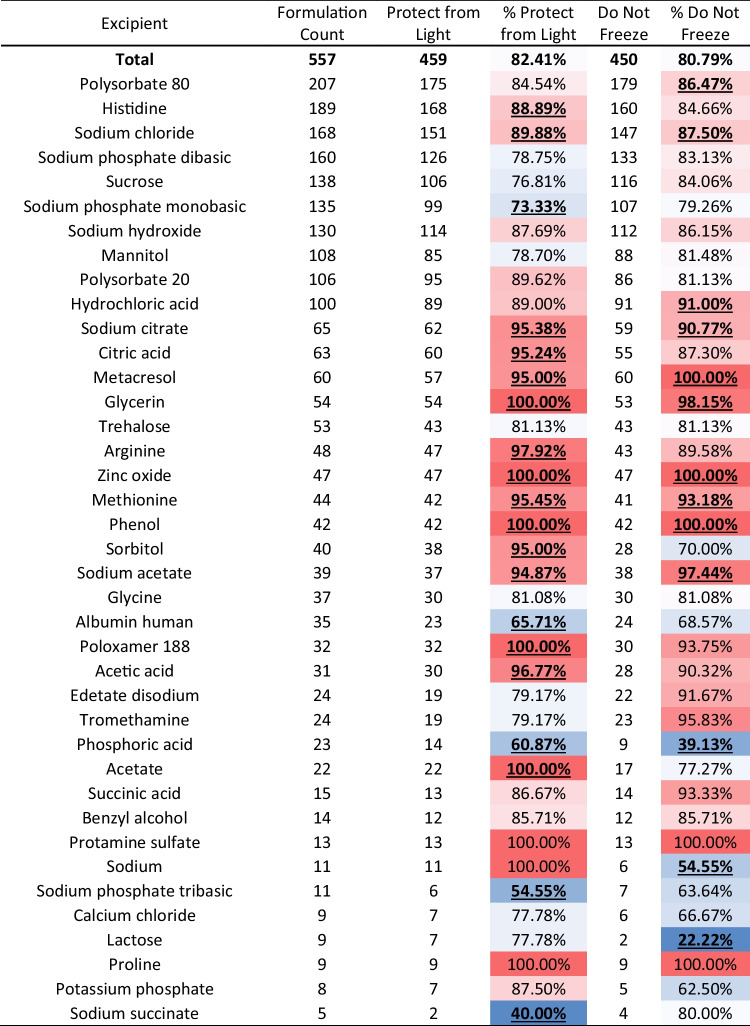
Number of Formulations by Excipient with Light and Temperature Sensitivity Indication

^*^ Bolded and underlined percentages indicate a significant change (p < 0.05 via a binomial test) from the average indication percentage (82.41% for light sensitivity and 80.79% for freezing sensitivity). For the heat map, red coded numbers are higher than the midpoint average and blue coded numbers are lower than their respective midpoint average

**Table XVIII:** Number of Formulations by pH range with Light and Temperature Sensitivity Indication

pH	Total Formulations	Indication			
		Protect from Light		Do not freeze	
		Indications	Percentage	Indications	Percentage
Total	352	325	92.33%	319	90.63%
pH 1–5.99	134	130	**97.01%**	121	90.30%
pH 6–8	217	194	89.40%	197	90.78%
pH 8.01–14	1	1	100.00%	1	100.00%

^*^ Bolded and underlined percentages indicate a significant change (p < 0.05 via a binomial test) from the average indication percentage (92.33% for light sensitivity and 90.63% for freezing sensitivity)

## Discussion

In this report, we provide a comprehensive survey of therapeutic protein drug formulations in the context of storage recommendations provided with light and temperature sensitivity indications. Storage conditions were assessed by multiple formulation attributes including API molecule types, dosage form, number of intended doses, container types, and diluents used for reconstitution and dilution if needed. These analyses provide an insight on the current recommendations made by the pharmaceutical industry for light and temperature exposure based on various formulations and molecule types.

A higher proportion of formulations manufactured as liquids have indications to protect from light and not to freeze when compared to formulations manufactured as lyophilized powders. This agrees with previous findings of increased overall stability of lyophilized biological samples versus their liquid counterparts [14–16]. A similar trend is observed when looking at container types. Products manufactured in autoinjectors, prefilled-syringes, and pens that mostly contain liquid formulations have a higher proportion of formulations with light and temperature sensitivity when compared to products in vials that are used for both lyophilized powder and liquid formulations. Our findings are also consistent with the findings of the University of Illinois previously acknowledging the light sensitivity of injectable drugs while creating a list of light sensitive drugs for clinical use [17]. The increased prevalence of light and temperature sensitivity could be attributed to the increase in liquid formulations in autoinjectors, pre-filled syringes, and pens. A lower proportion of products manufactured in bottles or tubes had light or temperature sensitivity, which contained solid and semisolid dosage forms that are traditionally considered more stable than liquid formulations [18].

Prevalence of light and temperature sensitivity was also compared among the different presentation statuses of products. It was observed that there were fewer indications or clear recommendations to protect from light or not to freeze for reconstituted or diluted formulations when compared to formulations that are used as-supplied, even though more formulations are allowed to be stored at room temperature, where light exposure is expected. Lack of light and temperature sensitivity on these reconstituted and diluted formulations may be related to their recommended storage time, which is relatively short compared to as-supplied presentations but could also represent an understudied aspect of biotechnology-derived therapeutic products and their stability upon reconstitution or dilution. Further studies are needed to determine whether there are other mechanistic, logistic or regulatory factors contributing to the lower prevalence of light and temperature sensitivity indications for reconstituted or diluted formulations [3].

The survey on excipients across formulations also enabled us to observe light and temperature sensitivity indications for formulations specifically designed with possible enhancement and/or protection of light and temperature-induced degradation. For example, when histidine is used as an excipient, it resulted in a higher incidence of light sensitivity which correlated well with a previous finding that histidine buffers can undergo photooxidation crosslinking to monoclonal antibodies [19]. Citrate buffers, which are the third most common buffer in monoclonal antibody products, were also found to have high incidences of protect from light designations, which is in agreement with studies that have shown photochemical degradation of citrate leading to carbonylation of therapeutic proteins [20, 21]. Although the database showed similar trends to effects observed in these studies, future work is needed to confirm whether other products containing these excipients display similar degradation pathways. Interestingly, sodium succinate and sodium phosphate buffers did not have a higher occurrence of do not freeze indications even though both buffer systems have been identified to undergo significant pH changes due to selective crystallization of buffer components when going through a freeze thaw cycle [22, 23]. Although the effects of sodium phosphate buffers on protein denaturation during freezing has been known since the early 2000’s it is still utilized as a buffering agent as of 2022 in some formulations. Additionally, succinic acid has been utilized in 15 formulations since 1999, the majority of which were approved between 2016 and 2021, while being attributed to pH swings during freezing since 2010 [24]. Acidic pH values were observed to be associated with higher than average light sensitivity. However, future research is needed to determine the molecular effects of pH on photo and freezing sensitivity to determine whether the effects are pH related or due to the fact that many buffers used to reach acidic pH also had higher than average photo sensitivity (acetate, citrate, and histidine). Additionally, glycerin was observed to have significantly higher rates of do-not-freeze indications even though it is generally regarded as a cryoprotectant of proteins [25]. Unexpectedly, formulations with polysorbate did not have significantly higher instances of recommendations for light sensitivity even though polysorbate has been found to affect the quality of monoclonal antibodies when exposed to light due to peroxide generation, pH changes, and changes in surface tension [13, 26, 27]. Although some of these excipients followed predictable trends in light and temperature sensitivity, these correlations have also highlighted some other excipients that warrant further investigation. For example, human serum albumin was observed to result in significantly lower protect from light indications when used as an excipient even though it has been shown to be photosensitive [28]. More studies could investigate whether excipients such as human serum albumin’s own photosensitivity is able to shield photooxidation of other biological materials via molecular crowding. With this data in hand, it is important to note a caveat that this work provides overall information on the specific correlation of certain excipients and their subsequent likelihood of being in a formulation of a drug product that is deemed sensitive to freezing or light. Intrinsic properties specific to each protein (e.g. concentration and amino acid content) can drive sensitivity to light and temperature but are not covered in this work.

We envision that our evidence-driven analyses can be further utilized in the research for relationships among different formulation attributes as well as assist drug developers and manufacturers in performing risk assessment and identifying any links between the risk and specific product attributes to develop stable therapeutic protein drugs. If one is to design protein products that are capable of consistent use and quality across different product presentation types and climate zones it will be critical to fully understand the variables that drive photo and thermal stability. As these data increase in utilization, photostability studies can be improved, biologic stabilizers can be further understood, and formulations that can withstand variability in storage conditions can be developed leading to safer and more stable biologic therapeutics.

## Data Availability

All datasets generated or analysed during this study are included in this published article.

## Abbreviations

(NDC): National Drug Code

## References

[1] Rao VA, Kim JJ, Patel DS, Rains K, Estoll CR (2020). A Comprehensive Scientific Survey of Excipients Used in Currently Marketed, Therapeutic Biological Drug Products. Pharm Res.

[2] Schoneich C (2020). Photo-Degradation of Therapeutic Proteins: Mechanistic Aspects. Pharm Res.

[3] International Conference on Harmonization (1997). Guidelines for the Photostability Testing of New Drug Substances and Products. Fed Reg..

[4] Kerwin BA, Remmele RL (2007). Protect from Light: Photodegradation and Protein Biologics. J Pharm Sci.

[5] Shah DD, Zhang J, Maity H, Mallela KMG (2018). Effect of photo-degradation on the structure, stability, aggregation, and function of an IgG1 monoclonal antibody. Int J Pharm.

[6] Boll B, Bessa J, Folzer E, Rios Quiroz A, Schmidt R, Bulau P (2017). Extensive Chemical Modifications in the Primary Protein Structure of IgG1 Subvisible Particles Are Necessary for Breaking Immune Tolerance. Mol Pharm.

[7] List of Approved NDAs for Biological Products That Were Deemed to be BLAs on March 23, 2020: U.S. Food and Drug Administration.; [Available from: https://www.fda.gov/media/119229/download.

[8] Purple Book: lists of licensed biological products with reference product exclusivity and biosimilarity or interchangeability evaluations.: U.S. Food and Drug Administration; Available from: https://purplebooksearch.fda.gov/.

[9] Drugs@FDA: U.S. Food and Drug Administration; [Available from: https://www.accessdata.fda.gov/scripts/cder/daf/.

[10] Stryjewska A, Kiepura K, Librowski T, Lochynski S (2013). Biotechnology and genetic engineering in the new drug development. Part I. DNA technology and recombinant proteins. Pharmacol Rep..

[11] Biosimilar and Interchangeable Products: U.S. Food & Drug Administration; updated December 13, 2022. Available from: https://www.fda.gov/drugs/therapeutic-biologics-applications-bla/biosimilars.

[12] Subelzu N, Schoneich C (2021). Pharmaceutical Excipients Enhance Iron-Dependent Photo-Degradation in Pharmaceutical Buffers by near UV and Visible Light: Tyrosine Modification by Reactions of the Antioxidant Methionine in Citrate Buffer. Pharm Res.

[13] Singh SR, Zhang J, O'Dell C, Hsieh MC, Goldstein J, Liu J (2012). Effect of polysorbate 80 quality on photostability of a monoclonal antibody. AAPS PharmSciTech.

[14] Molnar A, Lakat T, Hosszu A, Szebeni B, Balogh A, Orfi L (2021). Lyophilization and homogenization of biological samples improves reproducibility and reduces standard deviation in molecular biology techniques. Amino Acids.

[15] Pikal MJ, Rey L (2010). Mechanisms of Protein Stabilization During Freeze-Drying Storage: The Relative Importance of Thermodynamic Stabilization and Glassy State Relaxation Dynamics. Freeze-Drying/Lyophilization of Pharmaceutical and Biological Products.

[16] Yoneda S, Torisu T, Uchiyama S (2021). Development of syringes and vials for delivery of biologics: current challenges and innovative solutions. Expert Opin Drug Deliv.

[17] Ghaibi S, Ipema HJ, Soni R, Debartolo RJ, Mancuso CE (2014). Light-Sensitive Injectable Prescription Drugs. Hosp Pharm.

[18] Lajoinie A, Henin E, Kassai B, Terry D (2014). Solid oral forms availability in children: a cost saving investigation. Br J Clin Pharmacol.

[19] Powell T, Knight MJ, O’Hara J, Burkitt W (2020). Discovery of a Photoinduced Histidine-Histidine Cross-Link in an IgG4 Antibody. J Am Soc Mass Spectrom.

[20] Valliere-Douglass JF, Connell-Crowley L, Jensen R, Schnier PD, Trilisky E, Leith M (2010). Photochemical degradation of citrate buffers leads to covalent acetonation of recombinant protein therapeutics. Protein Sci.

[21] Strickley RG, Lambert WJ (2021). A review of Formulations of Commercially Available Antibodies. J Pharm Sci..

[22] Ukidve A, Rembert KB, Vanipenta R, Dorion P, Lafarguette P, McCoy T, et al. Succinate buffer in biologics products: real world formulation considerations, processing risks and mitigation strategies. J Pharm Sci. 2022;112(1):138–47. 10.1016/j.xphs.2022.05.026 35667631

[23] Pikal-Cleland KA, Rodríguez-Hornedo N, Amidon GL, Carpenter JF (2000). Protein Denaturation during Freezing and Thawing in Phosphate Buffer Systems: Monomeric and Tetrameric β-Galactosidase. Arch Biochem Biophys.

[24] Sundaramurthi P, Shalaev E, Suryanarayanan R (2010). Calorimetric and Diffractometric Evidence for the Sequential Crystallization of Buffer Components and the Consequential pH Swing in Frozen Solutions. J Phys Chem B.

[25] Strambini GB, Gabellieri E (1996). Proteins in frozen solutions: evidence of ice-induced partial unfolding. Biophys J.

[26] Donbrow M, Azaz E, Pillersdorf A (1978). Autoxidation of Polysorbates. J Pharm Sci.

[27] Prajapati I, Subelzu N, Zhang Y, Wu Y, Schöneich C (2022). Near UV and Visible Light Photo-Degradation Mechanisms in Citrate Buffer: One-Electron Reduction of Peptide and Protein Disulfides promotes Oxidation and Cis/Trans Isomerization of Unsaturated Fatty Acids of Polysorbate 80. J Pharm Sci.

[28] Pedersen AO, Schonheyder F, Brodersen R (1977). Photooxidation of Human Serum Albumin and Its Complex with Bilirubin. Eur J Biochem.

